# Effectiveness of drone-based thermal sensors in optimizing controlled environment agriculture performance under arid conditions

**DOI:** 10.1038/s41598-025-94432-0

**Published:** 2025-03-16

**Authors:** Rawan Al-Najadi, Yaseen Al-Mulla, Ibtisam Al-Abri, Abdullah Mohammed Al-Sadi

**Affiliations:** 1 Department of Soils, Water, and Agricultural Engineering, Sultan Qaboos University, 123 Al-Khod, Oman; 2 Remote Sensing and GIS Research Center, Sultan Qaboos University, 123 Al-Khod, Oman; 3 Department of Natural Resource Economics, Sultan Qaboos University, 123 Al-Khod, Oman; 4 Department of Plant Sciences, Sultan Qaboos University, 123 Al-Khod, Oman; 5 College of Agriculture, University of Al Dhaid, Al Dhaid, United Arab Emirates

**Keywords:** Controlled environment agriculture, Wireless sensor network, Drone, Remote sensing, Thermal images, Index relative stomatal conductance, Plant stress responses, Agroecology

## Abstract

Controlled environmental agriculture (CEA), integrated with internet of things and wireless sensor network (WSN) technologies, offers advanced tools for real-time monitoring and assessment of microclimate and plant health/stress. Drone applications have emerged as transformative technology with significant potential for CEA. However, adoption and practical implementation of such technologies remain limited, particularly in arid regions. Despite their advantages in agriculture, drones have yet to gain widespread utilization in CEA systems. This study investigates the effectiveness of drone-based thermal imaging (DBTI) in optimizing CEA performance and monitoring plant health under arid conditions. Several WSN sensors were deployed to track microclimatic variations within the CEA environment. A novel method was developed for assessing canopy temperature (Tc) using thermocouples and DBTI. The crop water stress index (CWSI) was computed based on Tc extracted from DBTI. Findings revealed that DBTI effectively distinguished between all treatments, with Tc detection exhibiting a strong correlation (R^2^ = 0.959) with sensor-based measurements. Results confirmed a direct relationship between CWSI and Tc, as well as a significant association between soil moisture content and CWSI. This research demonstrates that DBTI can enhance irrigation scheduling accuracy and provide precise evapotranspiration (ETc) estimates at specific spatiotemporal scales, contributing to improved water and food security.

## Introduction

There is a noticeable decline in agricultural land due to climate change and rising global consumption^1^. According to estimates from the UN Food and Agriculture Organization, the global population is expected to reach 10 billion by 2050 and 11.2 billion by 2100^2^. Controlled environment agriculture (CEA) is an effective method for increasing agricultural productivity, as it protects plants and optimizes growth conditions^3^. Greenhouses, a common form of CEA, allow farmers to cultivate any crop by regulating light, heat, humidity, and nutrients^1^, creating an optimal indoor environment for plant growth.

Climate variations, particularly in arid and semi-arid regions like Oman, hinder traditional agriculture and contribute to food shortages, posing future challenges^4,5^. The expansion of greenhouse production, especially in arid regions, is driven by the favorable climate in these areas compared to the extreme and unpredictable conditions caused by climate change^6^.

An automated CEA system regulates and monitors climate parameters using digital devices like sensors, ensuring optimal growth conditions^7^. The "third green revolution" refers to the integration of wireless sensors in smart agriculture, enhancing the efficiency of CEA production^8–11^.

Jiménez et al.^12^ implemented a wireless sensor network (WSN) infrastructure to effectively control microclimate parameters in CEA, providing an automated and efficient alternative to manual methods. Mohammed^13^ suggests that smart CEA can positively impact crop productivity by managing environmental factors such as temperature, light intensity, relative humidity, CO_2_ levels, and soil moisture. Continuous monitoring of plants and internal ecological conditions is essential for CEA, as noted in^14^. Al-Mulla et al.^6^ found that using sensors to monitor microclimate conditions in an evaporative-cooled controlled environment improved crop productivity and yield.

However, the implementation and adaptation of such technologies still require improvement, particularly in developing countries, where limitations can hinder agricultural development and overall food security. Additionally, despite their potential in agriculture, drone models are not yet widely adopted in CEAs. Research studies^14–17^ have identified several challenges associated with CEA in arid and semiarid regions. Among these challenges are nonuniform temperature and humidity distribution, which increases the risk of fungal disease outbreaks in crops. Moreover, inadequate ventilation and cooling can create unfavorable environmental conditions, raising crop evapotranspiration demand and leading to inefficient irrigation water use. Effective water management and irrigation technologies are essential, but careful planning is required due to high ambient temperatures. Implementing cooling systems, such as evaporative cooling and shading techniques, is crucial for crop growth in arid environments.

Conversely, many researchers have utilized drones, or unmanned aerial vehicles (UAVs), in external agricultural fields for various applications, including monitoring crop development over time and assessing plant health^18–25^. Some studies have also explored drone applications in controlled environments, primarily for monitoring CEA microclimates and greenhouse pollination^26–28^.

Komarchuk et al.^28^ examined the use of drones in industrial greenhouses to optimize operations and improve monitoring, data collection, and environmental management, addressing challenges related to efficiency, automation, and advanced imaging and sensing technologies. According to^29,30^, drones can rapidly collect the data needed to monitor plant conditions and environmental factors in Dutch greenhouses. However, the accuracy of environmental monitoring using drones can vary based on equipment calibration, methodology, and sensor precision. Heemskerk et al.^27^ found drone-based scouting to be 96% effective in detecting plant diseases, surpassing human scouting, which achieved 70% accuracy.

None of the studies mentioned above have incorporated emerging technologies such as UAV-based thermal imaging. Moreover, it remains unclear whether UAV applications in CEA can be adapted to hyper-arid and semiarid conditions similar to those in Oman. This research aimed to evaluate the effectiveness of remotely sensed plant thermal characteristics, captured via drones, in enhancing CEA performance and enabling continuous plant health monitoring. The novelty of this study lies in the significant application of UAV technology in CEA, particularly for plant-based monitoring of growing conditions. Various plant growth metrics can be obtained through imaging techniques, offering valuable insights into plant development. Thus, this study focused on assessing the effectiveness of drone-based thermal imaging in improving CEA performance and monitoring pepper plant health through the following subobjectives: (a) Monitoring the microclimate and soil moisture within the CEA, (b) Developing algorithms for WSN-based sensors, (c) Implementing different irrigation schemes ranging from under- to over-irrigation, and (d) Detecting canopy surface temperature in real-time using drone-based thermal imaging and validating it through in-situ measurements.

## Material and methods

**Fig. 1:**
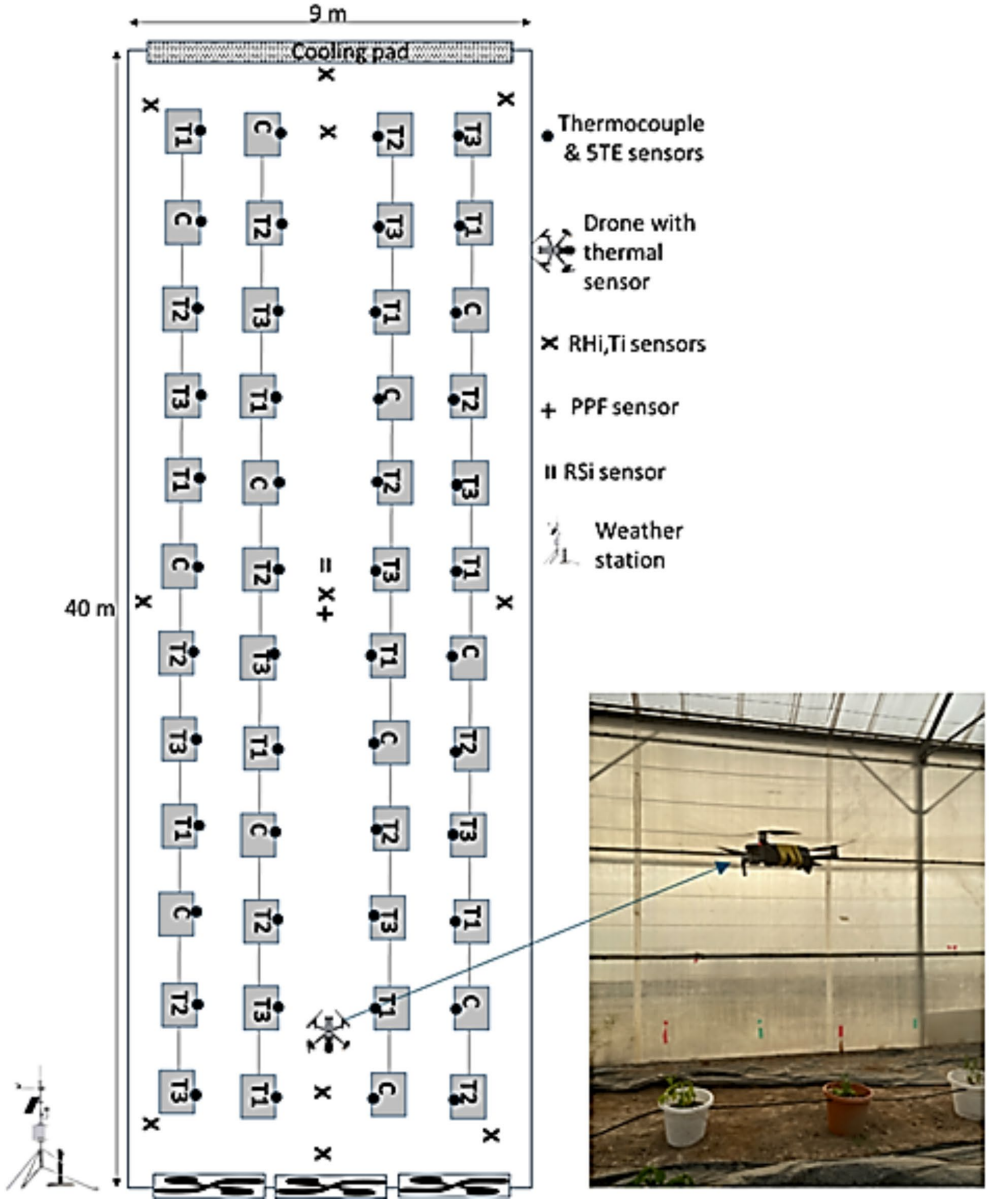
Experimental setup of the sensors inside the controlled environmental agriculture (CEA) structure and the drone equipped with a thermal camera.

The experiment was conducted at Sultan Qaboos University in Oman (23° 36.015′ N, 58° 10.044′ E, 47 m above mean sea level) using an arch-type CEA structure measuring 9 m in width, 40 m in length, and 6 m in ridge height. The total ground area of the structure was 360 m^2^, with a volume of 2160 m^3^. The structure was covered with a 200 µm thick polyethylene film (LDPE, Plastika Kritis, Iraklion Crete, Greece) and equipped with a pad-and-fan evaporative cooling system. This system included a cross-fluted cellulose pad (CELdek 7090-15 type, Munters, Kista, Sweden) measuring 6.6 m in width, 1.8 m in height, and 0.11 m in thickness, installed at the north end. Three fans (1.5 hp each, with a total capacity of 4000 m^3^ h^−1^) were installed at the south end, opposite the wet pad, drawing air from the greenhouse to the outside. The fans were activated whenever the internal air temperature exceeded 28 °C. Pepper plants were cultivated in the CEA during both summer and winter seasons. The plants were distributed in 48 pots arranged in 12 rows and four columns, with one plant per pot. An insecticide was applied before transplantation, and the soil consisted of a mixture of peat moss and sand. A drip irrigation system was used to apply four irrigation levels based on crop water requirement (ETc) twice daily. One irrigation level, set at 100% ETc, served as the control (C) since it provided the exact water requirement. An under-irrigation treatment (T1) was applied at 50% ETc, while two over-irrigation treatments were applied at 150% ETc (T2) and 200% ETc (T3). All treatments and the control were conducted with 12 replicates in a randomized complete block design (Table 1; Fig. 1).

**Table 1:** Tested treatements and their corresponding irrigation applications.

Treatment number	Irrigation level	Number of drippers per pot	ETc ${(\text{mm day}}^{-1}$)	Emitter Discharge (L h^−1^)
T1	50% ETc	1	7.3	4.2
C	100% ETc	2	14.6	8.4
T2	150% ETc	3	21.9	12.6
T3	200% ETc	4	29.3	16.8

The ambient weather conditions were monitored and recorded using a nearby weather station located 400 m from the CEA structure. This station was equipped with HMP45C sensors (Campbell Scientific, Logan, Utah) to measure air temperature (T_o_, °C) and relative humidity (RH_o_, %), a CM-3 pyranometer (Kipp & Zonen, Delft, The Netherlands) to measure incoming solar radiation (RS_o_, W/m^2^), and a 014A cup anemometer (Campbell Scientific, Logan, Utah) to measure wind speed (U, m/s) and direction (WD).

Inside the CEA, climate conditions were monitored using HMP45C and HC2S3-L sensors (Campbell Scientific, Logan, Utah) to measure air temperature (Ti, °C) and relative humidity (RHi, %). These sensors were distributed across 11 locations: three were placed diagonally around the pots, three near the west wall, three near the east wall, and the remaining two near the fan and the cooling pad. All sensors were installed at a height of 3 m above the ground. The collected data were averaged and recorded every 30 min using CR1000 data loggers (Campbell Scientific, Logan, Utah).

The measured indoor air temperature and relative humidity were used to calculate the atmospheric vapor pressure deficit (VPD, kPa) based on^31^ using the following equation:

1 $$\text{VPD }= 0.6108 {e}^{\frac{17.27{T}_{i}}{{T}_{i}+37.3}}(1-{\text{RH}}_{i})$$

A CM-3 pyranometer (Kipp & Zonen, Delft, The Netherlands) was installed just below the cover material to measure the solar radiation (RSi, W/m^2^) inside the CEA structure. Both RSi and RSo measurements were used to calculate the transmissivity (τ) of the cover material using the following equation:

2 $$\uptau = \frac{{RS}_{i}}{{RS}_{o}}$$

An MQ-500 full-spectrum quantum photosynthetically active radiation sensor (Apogee, USA) was installed above the plant canopy to measure the photosynthetic photon flux density (PPF, µmol m^−2^ s^−1^) within the 400–700 nm range.

Soil moisture content was measured using 5TE sensors (Meter Group, Washington, USA) to determine the volumetric water content for each treatment. These sensors were installed at a depth of 10 cm and distributed throughout the greenhouse, as illustrated in Fig. 1s. The readings were recorded every 60 min using the ECH2O Utility data logger. The sensor-based canopy temperature (Tcs, °C) was measured using 48 thermocouples (type T, wire diameter of 0.5 mm, Omega Engineering Ltd., Manchester, U.K.), which were taped to the undersides of the leaves of each of the 48 plants (Fig. 1). A CR3000 data logger (Campbell Scientific, Logan, Utah) was programmed to record the average thermocouple readings every 30 min using PC 200W version 4.5 software.

A thermal imaging camera (FLIR One Pro, Teledyne FLIR, USA) was mounted on a drone (Mavic 2 Pro, DJI, China) and calibrated using a reference point with a known temperature, following the calibration process described by García-Tejero et al.^32^. Thermal imagery was conducted by flying the drone at a steady altitude of 2 m above the plant canopy, capturing images three times daily: at 8:00 AM, 12:00 noon, and 4:00 PM. A total of 400 thermal images were collected in 14-bit JPG format and registered using the Thermal MSX® mode. All thermal images of the plant canopy were processed using Thermal Studio Pro software, which featured a spot meter for automated analysis. This advanced technology enabled the generation of comprehensive thermal images combined with visible light elements, allowing for quick identification of problematic heat patterns.

The relationship between plant water stress and canopy temperature from thermal images was assessed using the crop water stress index (CWSI)^26,31^. Air temperature (T_a_, °C) was compared to canopy temperature obtained from thermal images (Tcm, °C) using the following equation:

3 $$CWSI = \frac{Tcm-Tcmmin}{Tcmmax-Tcmmin}-\frac{Ti-Timin}{Timax-Timin}$$

In this study, the canopy temperature extracted from thermal imagery was used instead of conventional in-situ thermocouple sensor-based measurements. This approach significantly reduced costs compared to the conventional method. In this context, Tcm, Tcmmax, and Tcmmin represent the average, maximum, and minimum canopy temperatures derived from thermal images (°C), respectively, while Ti, Timax, and Timin denote the average, maximum, and minimum inside air temperatures (°C).

Another approach to measuring plant water stress is the determination of relative stomatal conductance (Ig, dimensionless). Stomatal conductance plays a crucial role in plant physiological processes, as it regulates gas exchange through photosynthesis and transpiration, maintaining the balance of carbon and water content^28,33^. According to^34^, stomatal conductance to water is significantly correlated with canopy temperature and was calculated using the following equation:

4 $${l}_{g} = \frac{(Tdry-Tcmax)}{(Tcmin-Twet)}$$

For this equation, the canopy temperature extracted from thermal imagery was used instead of conventional in-situ thermocouple sensor-based measurements, effectively reducing costs associated with the conventional method. In this context, Twet and Tdry, which represent the maximum and minimum canopy temperatures, were determined by Tc_min_ and Tc_max_ (°C), respectively.

Statistical significance was assessed using analysis of variance (ANOVA) in JMP, Excel, and R Studio commercial software. Correlations, the coefficient of determination (R^2^), and probability (P) values were determined at a 5% significance level.

**Fig. 2:**
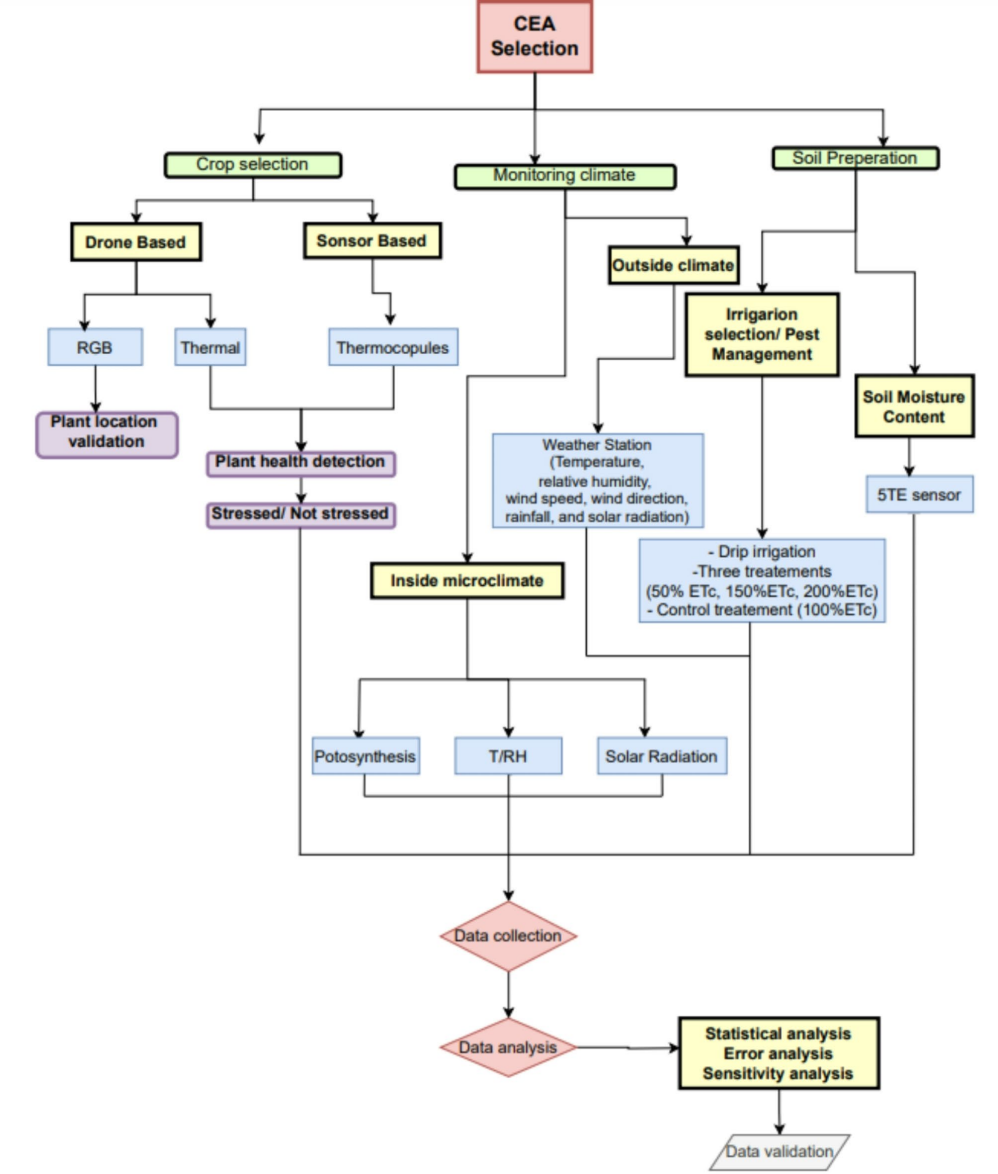
Flowchart illustrating the methodology of the study.

Table 2 summarizes the collected data and its characteristics, while Fig. 2 presents an architecture diagram illustrating the methodological workflow.

**Table 2:** Methodology for data collection.

Data type	Instrumentation	Characteristics
Drone-based Canopy Temperature	A FLIR ONE camera (FLIR® Systems, Inc., Wilsonville, OR USA)	Capture thermal images by detecting the canopy temperature with accuracy (± 3 °C). Measurements were taken three times, in the early morning, midday, and evening, to assess temperature variations and plant stress. The data were transferred to the FLIR Thermal Studio software
Sensor-based Canopy Temperature	Type T thermocouple sensors (Omega Engineering, Inc., CT, USA)	Measure the leaf surface temperature (Tc, ◦C) with accuracy (± 1 °C). The sensor was attached to the leaves of the plants. The data was collected through a data logger every 30 min taking the average
Inside Air Temperature	(HMP45C; Vaisala et al. /HC2S3-L; HYGROMER® et al.)	Distributed throughout the CEA to monitor the T. All the measurements were set to take data every 30 min, and the sensors were placed at a height of approximately 3 m. Data were collected using a data logger
Inside Relative Humidity	(HMP45C/HC2S3-L)	Distributed throughout the CEA to monitor the RH. All the measurements were set to take data every 30 min, and the sensors were placed at a height of approximately 3 m. Data were collected using a data logger
Inside Solar radiation (Light intensity)	Pyranometer (model CM3, Campbell Scientific, INC.)	It measures the total solar radiation to determine the roofing material’s optical transmission. It is installed at a height of approximately 5 m above the ground near the roof of the CEA. The data were taken on average every 30 min
Photosynthetically active radiation (PAR)	(Apogee, USA)	Determine the amount of light plants obtained. The spectral range is approximately 400–700 nm. It is used as a quantum light sensor to measure the light intensity within that band. The sensor was installed above the plants
Soil Moisture Content	5TE (Meter Group, Washington, USA)	Measure the soil’s volumetric water content for each treatment with a depth of 10 cm and frequency of 1 h for data collection
Ambient Weather Conditions	Weather station (temperature, relative humidity, wind speed, wind direction, rainfall, and solar radiation.)	Outside climatic data were obtained from a complete and automated weather station installed inside the research station. Records the environmental factors

### Error analysis

To analyze the error between the drone thermal imaging technology and the thermocouple sensors and evaluate their impact on the research results, errors were calculated using Eqs. (5), (6), and (7).

5 $${\text{Absolute}} \;{\text{Error}} = \left| {{\text{Tcm}} - {\text{Tcs}}} \right|$$

6 $${\text{Mean }}\;{\text{Absolute}} \;{\text{Error}} \left( {{\text{MAE}}} \right) = \frac{1}{{\text{N}}}\mathop \sum \limits_{i = 1}^{{\text{N}}} \left| { {\text{Tcmi}} - {\text{Tcsi}}} \right|$$

7 $${\text{Root}} \;{\text{Mean}} \;{\text{Square}}\; {\text{Error}} \left( {{\text{RMSE}}} \right) = \sqrt {\frac{{\mathop \sum \nolimits_{i = 1}^{N} \left( {{\text{Tcm}},{\text{i}} - {\text{Tcs}},{\text{i}}} \right)2}}{{\text{N}}}}$$

where Tcm is the thermal canopy temperature from a drone (°C), Tcs is the canopy temperature from the thermocouple sensors (°C), and i is the reading number.

## Results

### Thermal images acquisition and temperature extraction

**Fig. 3:**
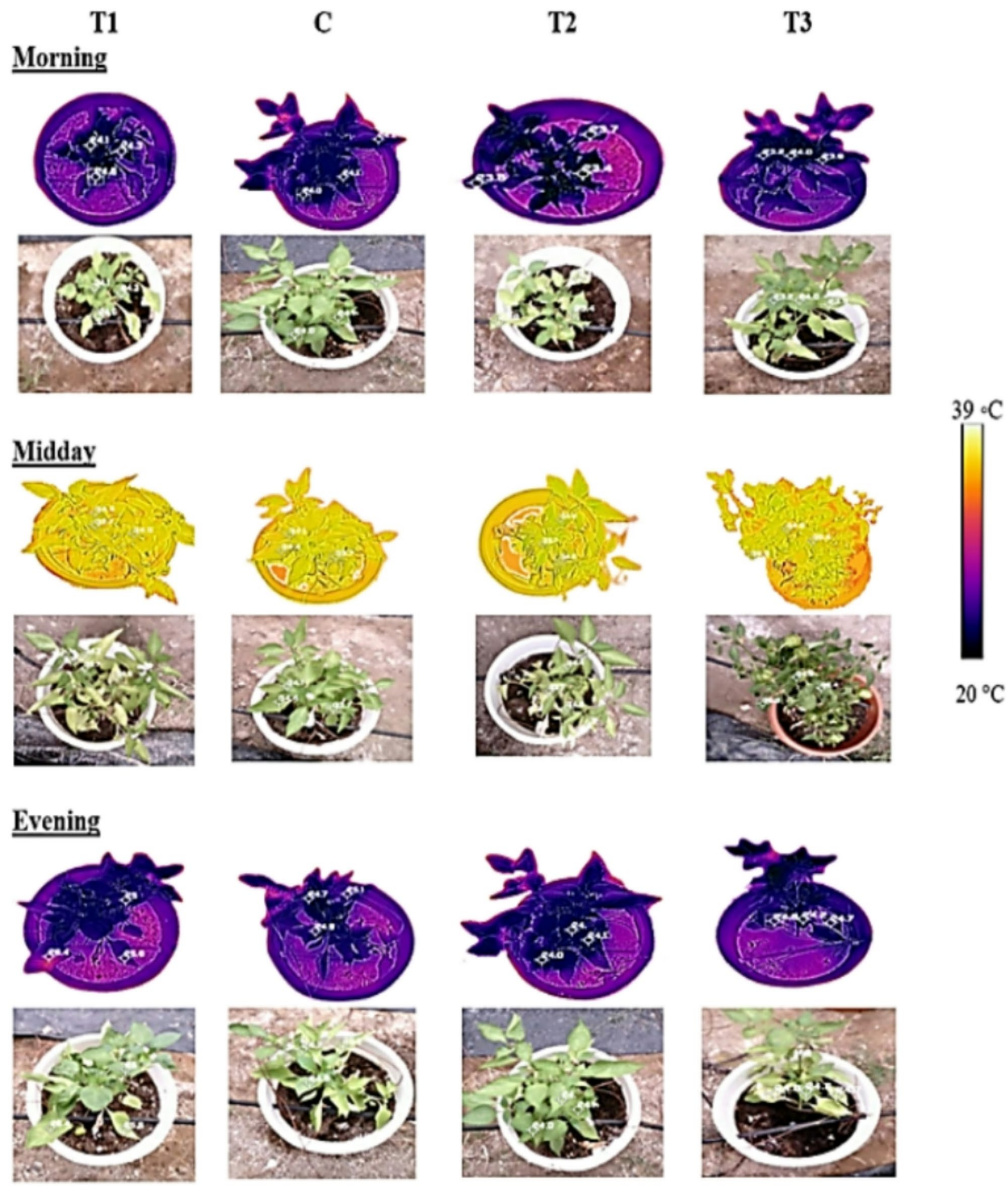
Thermal and visible images of pepper plants at different times: (**a**) morning, (**b**) midday, and (**c**) evening for various treatments. T1 represents under-irrigation (50% ETc), C is the control treatment (100% ETc), T2 corresponds to 150% ETc, and T3 represents over-irrigation (200% ETc).

**Fig. 4:**
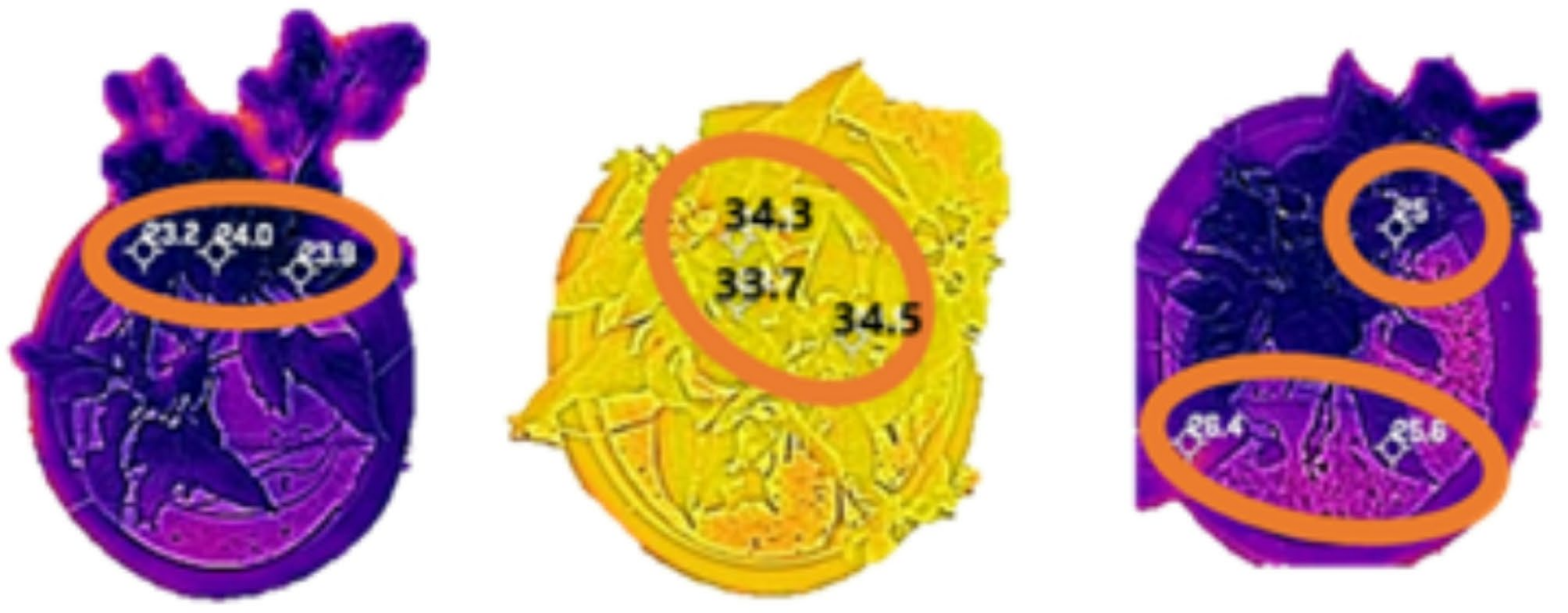
Extraction of canopy temperature (Tc).

**Fig. 5:**
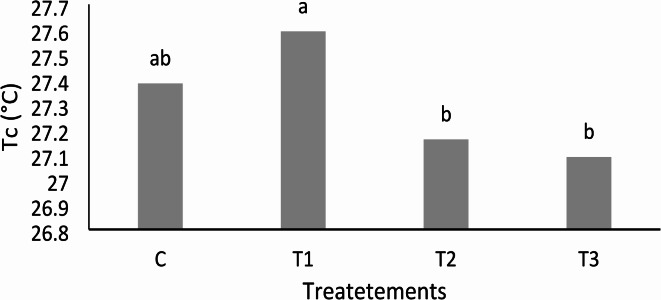
Effect of different treatment T1 (under irrigation, 50% ETc), C (control, 100% ETc), T2 (150% ETc), and T3 (over-irrigation, 200% ETc)—on canopy temperature (Tc) of pepper plants during daytime. Different capital letters indicate significant differences (Tukey’s test, *p* < 0.05, Mean (n = 12)).

**Fig. 6:**
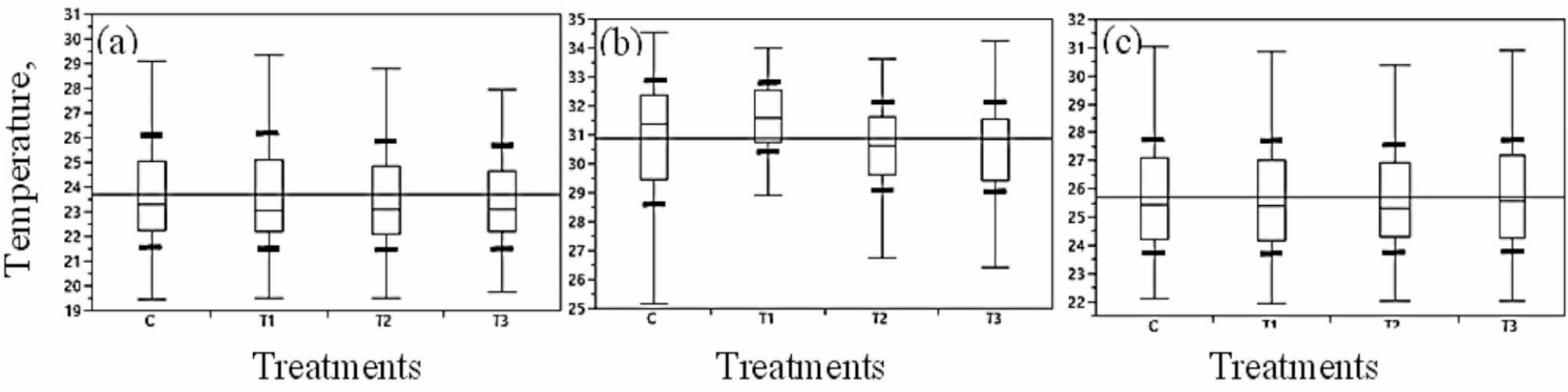
Box plots showing the average canopy temperature (Tc) data for each treatment: T1 (under irrigation, 50% ETc), C (control, 100% ETc), T2 (150% ETc), and T3 (over-irrigation, 200% ETc) at three time points: (**a**) morning, (**b**) midday, and (**c**) evening. Bold lines represent standard deviation (SD) lines, while horizontal lines indicate overall means.

The thermal and digital images obtained from the thermal camera are shown in Fig. 3, while Fig. 4 illustrates how Tc was extracted from the plants after processing the thermal images. The first treatment (T1) maintained low soil moisture, leading to reduced water absorption and higher canopy temperatures (Tc), indicating plant stress. ANOVA statistical analysis was conducted to examine the effects of the three treatments (T1, T2, and T3) and the control (C) on Tc. The results revealed a significant difference in Tc between treatments, with a notable effect on the C compared to T1, T2, and T3 (Fig. 5). Pairwise comparisons of the control (C) with the other treatments (T1, T2, and T3) indicated significant differences, particularly between C-T2, C-T3, and C-T1. However, the morning analysis showed no significant differences in canopy temperature across all treatments, indicating convergence in the early hours. Additionally, no significant differences in temperature were observed between treatments ranging from 23 to 25 °C, suggesting that morning irrigation maintained average canopy temperatures below 26 °C (Fig. 6a). By midday, Fig. 6b shows variations in temperature distributions among treatments, but no significant effect was found. The second irrigation session at 4 PM, coupled with reduced light intensity and air temperature, facilitated the recovery of Tc across treatments. However, no significant differences in temperatures between treatments were observed during this period (Fig. 6c).

### In-situ versus thermal measurements

**Fig. 7:**
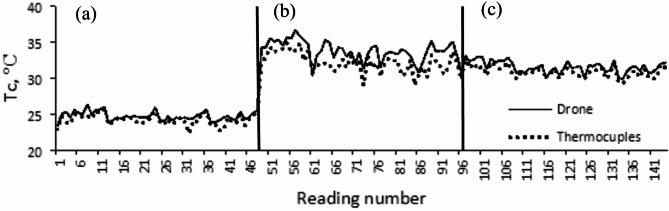
Trends of average canopy temperature (Tc) measured via thermocouple and drone drone-based thermal sensor at (**a**) morning, (**b**) midday, and (**c**) evening.

Figure 7 presents 144 temperature readings obtained from 48 direct thermocouple sensors and a wireless sensor via drone-based thermal imagery, recorded at three different times of the day: morning, midday, and evening. The figure indicates a similar trend between temperature measurements from the drone and direct sensors. In the morning, the readings were closely aligned, whereas the midday readings exhibited greater deviations. By the evening, the readings returned to patterns similar to those observed in the morning, showing a higher correlation between the two sensor types. The midday analysis suggests potential challenges in using a wireless thermal sensor to measure leaf canopy temperature during this period. High temperatures and intense solar radiation could impact the reflection intensity from plant leaves, affecting the sensor’s sensitivity. Additionally, the observed temperature differences may be attributed to the irrigation schedule, as no irrigation occurred at midday, with watering restricted to the morning and evening sessions.

**Fig. 8:**
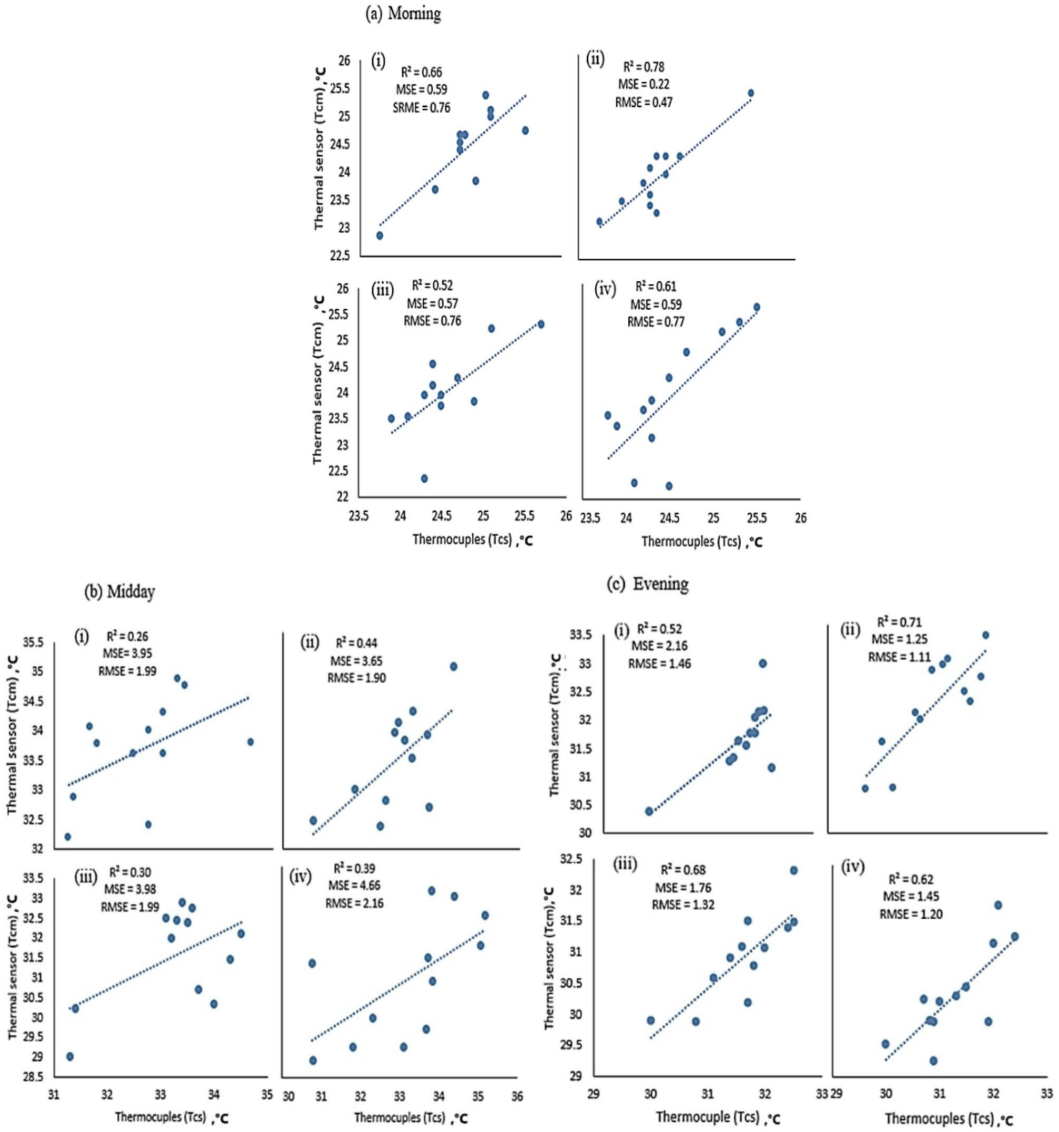
Canopy temperature measurements using thermocouples (Tcs in °C) versus drone-based thermal sensors (Tcm in °C) for each treatment: (i) T1, (ii) C, (iii) T2, and (iv) T3, recorded at (**a**) morning (8:00 a.m.), (**b**) midday (12:00 p.m.), and (**c**) afternoon (4:00 p.m.).The figure includes R^2^ (coefficient of determination) and error analysis using mean square error (MSE) and root mean square error (RMSE).

**Fig. 9:**
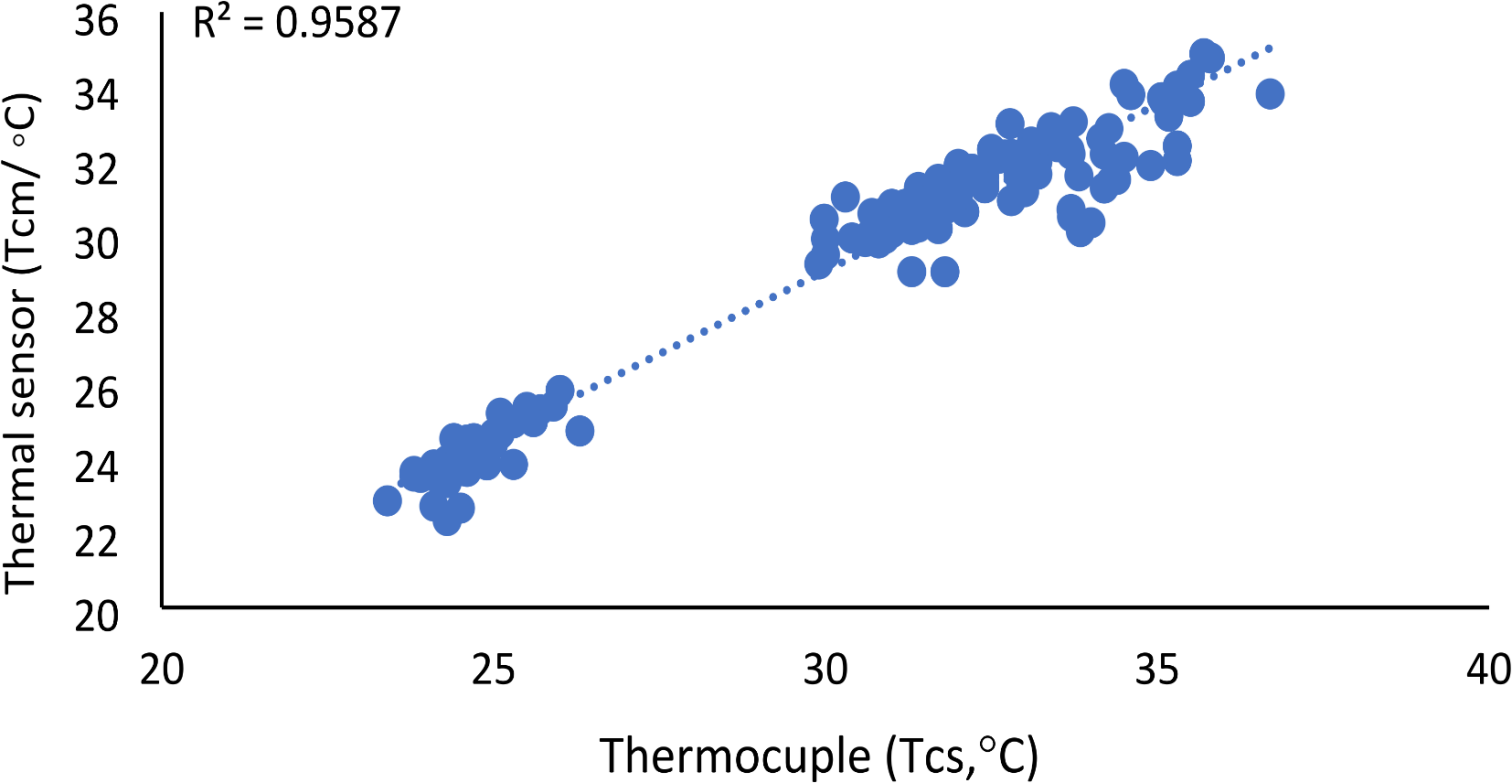
Overall correlation of canopy temperature measurements between the drone-based thermal wireless sensor (Tcm) and the thermocouple-based sensor (Tcs).

To investigate the similarity of Tc measurements between direct (thermocouple) and wireless (thermal) sensors across all treatments, Fig. 8 presents the analyses using a correlation relationship and the error analysis results calculated with Eqs. (4–6) for different time points. Additionally, Table 3 highlights the statistical analysis using ANOVA. The error analysis revealed discrepancies in Tc measurements across treatments (T1, T2, T3) and different times of the day. Errors were minimal in the morning, with MSE values of 0.59, 0.57, and 0.59, but slightly higher in the evening, with MSE values of 2.16, 1.76, and 1.25 for T1, T2, and T3, respectively. However, higher Tc values recorded at midday resulted in the highest errors, with MSE values of 3.98, 3.98, and 4.66 for T1, T2, and T3, respectively. This was due to sensor sensitivity differences at this time of day, where thermocouples ranged around ± 1 °C while drones exhibited ± 3 °C, leading to a consistent ± 2 °C variation in canopy temperatures. Moreover, the discrepancy between thermocouple-based and drone-based thermal measurements was attributed to the broader area captured by the drone’s thermal sensor, which was influenced by greenhouse surface thermal radiation, where reflective interference intensified under midday heat. Additionally, surface water evaporation during midday affected thermal readings, creating divergence between the localized thermocouple and the drone’s wider field of view. By synthesizing insights from Fig. 8 and Table 3, it is evident that all treatments showed no significant differences (*p*-value > 0.05) in the morning when comparing the two methods. Furthermore, the correlation plot in Fig. 8a revealed strong relationships between the methods, with an R^2^ exceeding 50% across all treatments. As expected, at midday, when the sun shines directly on the greenhouse roof with high radiation levels, a significant difference emerged, as shown in Fig. 8b. Correspondingly, R^2^ values in Fig. 8b were lower than in the morning. Additionally, the plot verifies that all treatments, except for C, were significant (*p*-value < 0.05). Figure 8c illustrates that the two platforms remained compatible in the evening when sunlight was minimal, although minor variations persisted. Nevertheless, an excellent correlation was observed between in-situ thermocouple sensors and drone-based thermal imaging, with an R^2^ value of 0.959 (Fig. 9).

**Table 3:** Adjustment equations, coefficients of determination (R^2^), and probability (*p*) values of ANOVA for Tc measurements using different methods in this study.

Treatments at morrning				
C	y = 0.9006x + 2.0372	R^2^ = 0.7754	P = 0.061655	n = 12
T1	y = 0.8x + 4.3469	R^2^ = 0.6625	P = 0.056713	n = 12
T2	y = 1.1839x—5.0573	R^2^ = 0.5197	P = 0.056612	n = 12
T3	y = 1.1947x—5.3532	R^2^ = 0.6103	P = 0.054059	n = 12
Treatments at midday				
C	y = 0.5736x + 13.072	R^2^ = 0.4403	P = 0.011086	n = 12
T1	y = 0.5939x + 12.778	R^2^ = 0.2606	P = 0.000102	n = 12
T2	y = 0.6776x + 9.0146	R^2^ = 0.3034	P = 0.00108	n = 12
T3	y = 0.4181x + 16.254	R^2^ = 0.3864	P = 0.003883	n = 12
Treatments at evening				
C	y = 0.6129x + 11.89	R^2^ = 0.7088	P = 0.09	n = 12
T1	y = 0.8441x + 4.275	R^2^ = 0.5202	P = 0.002938	n = 12
T2	y = 0.6129x + 11.89	R^2^ = 0.6774	P = 0.021511	n = 12
T3	y = 0.7158x + 7.7708	R^2^ = 0.6068	P = 0.038698	n = 12

### Thermal measurements versus environmental factors

**Fig. 10:**
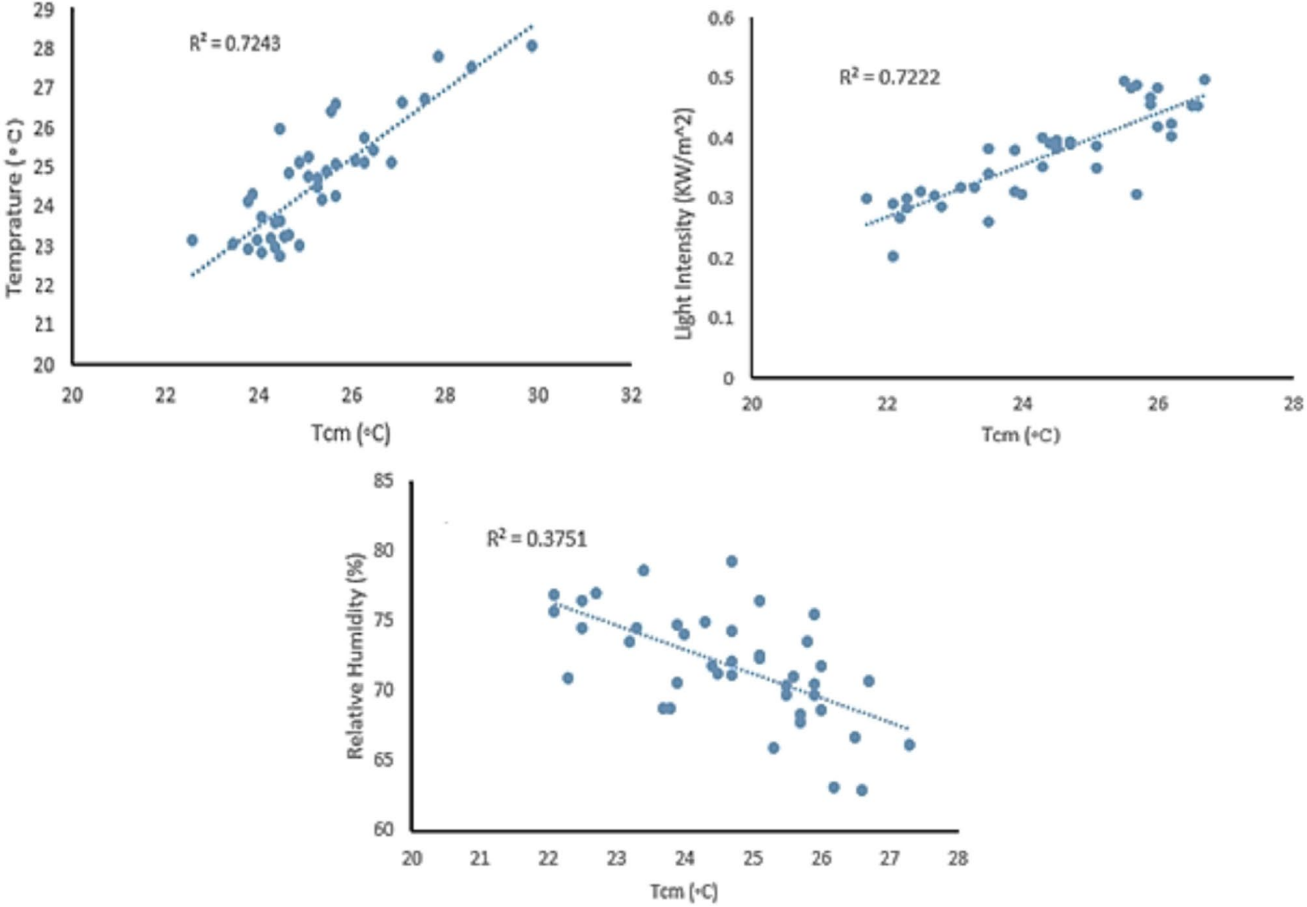
Correlation between canopy temperature measured by the drone-based thermal sensor (Tcm) and environmental factors, including light intensity, relative humidity, and air temperature.

A relationship was identified between the canopy temperature measured by the drone-based thermal sensor (Tcm) and environmental factors such as relative humidity, air temperature, and light intensity—inside a greenhouse, as shown in Fig. 10. A strong direct relationship was observed between Tcm and both inside air temperature (R^2^ = 0.7245) and light intensity (R^2^ = 0.7222). In contrast, an inverse but weaker correlation was found between Tcm and inside relative humidity, with R^2^ = 0.3751. These results indicate that thermal imagery can be influenced by environmental factors, particularly light intensity and air temperature.

### Thermal imaging-based CWSI

**Fig. 11:**
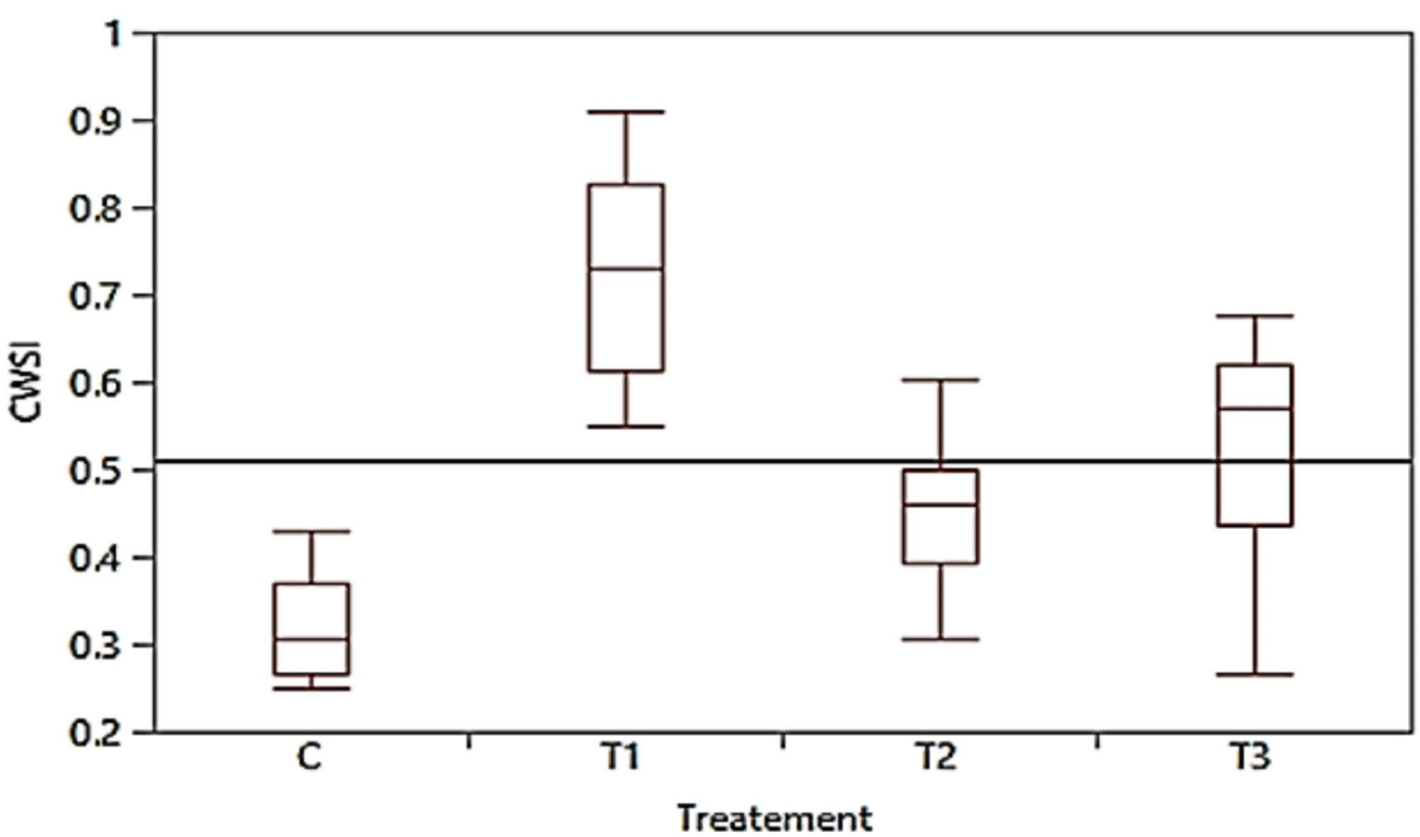
Calculated crop water stress index (CWSI) based on canopy temperature from thermal images (Tcm) for all treatments (T1, T2, T3, and C).

**Fig. 12:**
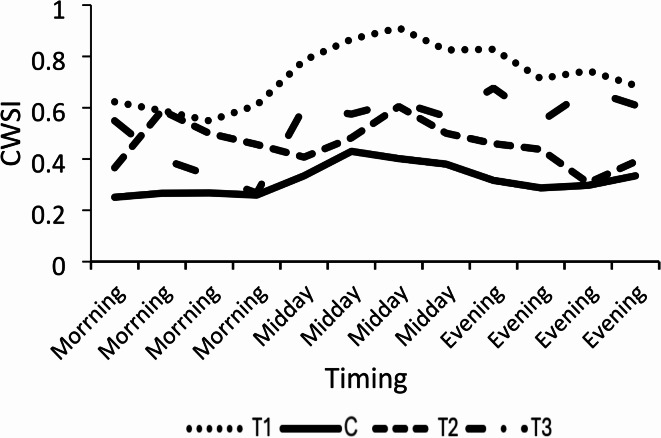
Behavior of crop water stress index (CWSI) for each treatment (T1, C, T2, and T3) at three different time points: morning, midday, and evening.

The use of drone-based thermal imaging proved effective in detecting plant water stress conditions by applying the thermal image-based temperature in the CWSI equation. Figure 11 illustrates how accurately drone-based thermal imaging reflected the actual water stress conditions of the plants. The line chart of plants under different treatments is shown in Fig. 12. The first treatment exhibited significant water stress, with a CWSI > 0.9. Initially, the plants resisted water deficiency, but over time, T1 plants became overstressed, displaying the highest CWSI values among all treatments. Conversely, the control treatment had the lowest CWSI, indicating it was the most suitable for plant growth. However, T2 and T3 plants occasionally showed high CWSI values due to over-irrigation, suggesting that some plants struggled to tolerate stress in both treatments. The highest CWSI values were recorded in all treatments at midday, as shown in Fig. 11.

### CWSI and stomatal conductance (Ig)

**Fig. 13:**
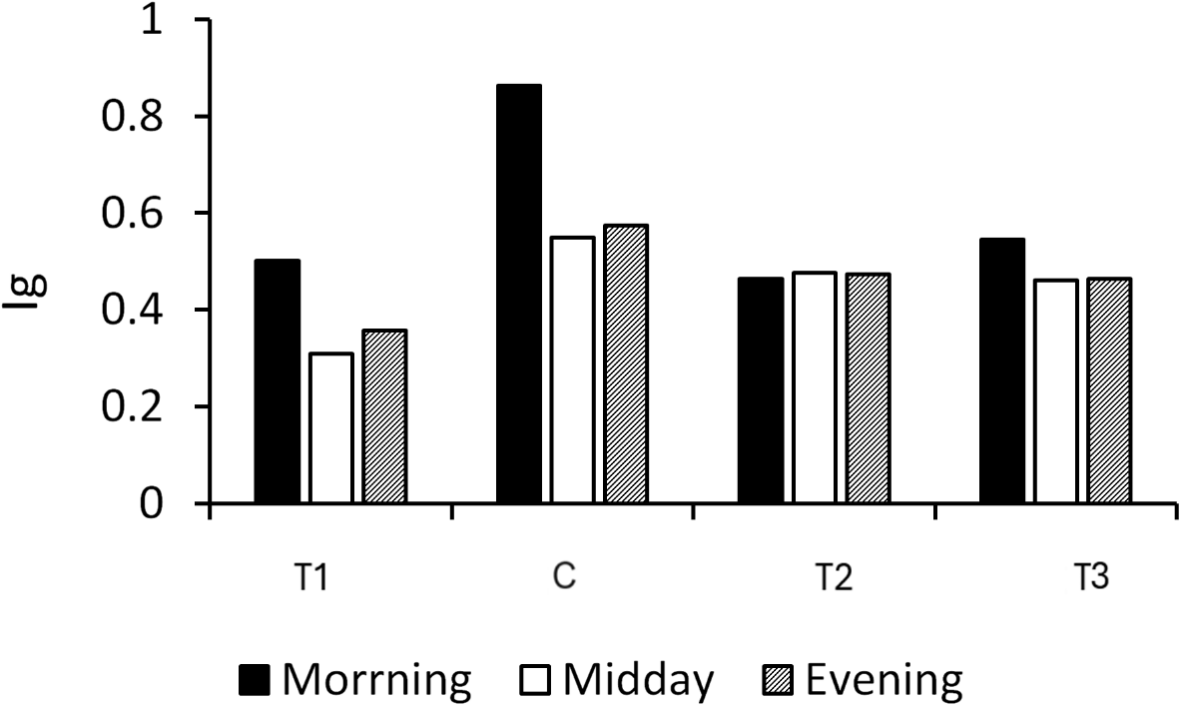
Relative stomatal conductance index (Ig) for the treatments (T1, T2, and T3) and the control (C) at morning, midday, and evening.

**Fig. 14:**
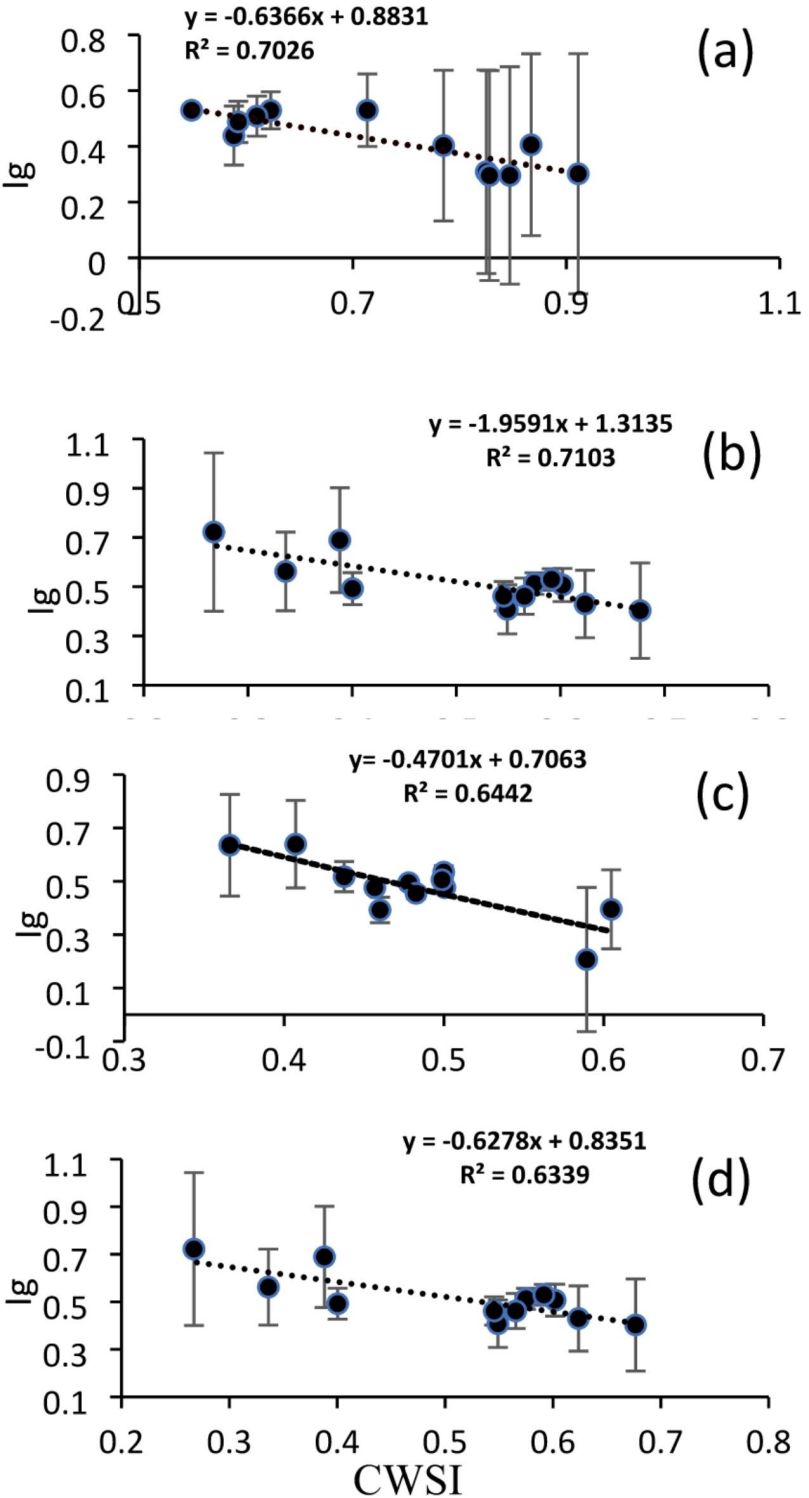
Correlation between the relative stomatal conductance index (Ig) and the crop water stress index (CWSI) for the treatments: (**a**) T1, (**b**) C, (**c**) T2, and (**d**) T3.

The lowest calculated Ig was recorded at midday in T1 (0.30) due to water scarcity and closed stomata (Fig. 13). In contrast, the morning timing showed the highest Ig value of 0.86 in the C treatment, indicating fully open stomata for maximum gas exchange and water vapor loss, which is typically associated with well-watered plants and optimal environmental conditions for plant growth. Plant water stress responses can be better understood by examining the correlation between Ig and CWSI. Stomatal conductance, which measures the amount of water lost through transpiration, has an inverse relationship with CWSI, which is derived from canopy temperature measurements. As shown in Fig. 14, increased CWSI leads to decreased Ig, indicating that stress can hinder stomatal opening, thereby affecting plant activity. The sensitivity of these indicators in identifying water stress levels is demonstrated by analyzing this relationship under various irrigation regimes and environmental conditions. The study found no significant differences in Ig values between treatments T2 and T3, with moderate correlations of R^2^ = 0.644 and 0.634, respectively. Additionally, a strong correlation was found between T1 and C, with R^2^ values of 0.7026 and 0.7103, respectively (Fig. 14). However, C was identified as the optimal treatment due to its ability to achieve the highest Ig values.

#### Model validation

Table 4 presents a model validation for plant water stress monitoring (Tcm) using drone thermal imaging, ensuring that the model accurately reflects real-world conditions. This process involves comparing thermal imaging data, including canopy temperature and derived CWSI values, against stomatal conductance under varying irrigation treatments, as shown in Fig. 14. High correlation coefficients between drone-derived metrics and physiological indicators confirm the model’s reliability. A plant water stress monitoring model was established based on drone thermal imaging data and validated to enhance its accuracy and reliability, using data from Fig. 12 and equations for each treatment (observed) along with Eq. (4) (predicted).

**Table 4:** Model validation for plant water stress monitoring based on thermal imaging temperature (Tcm) for all treatments.

CWSI	Ig observed from graph T1	Ig Predict from the Equation T1
0.6	0.529	0.501
0.75	0.402	0.406
0.9	0.301	0.246

CWSI	Ig Observed from graph C	Ig Predict from the Equation C
0.2	0.899	0.921
0.3	0.72577	0.626702
0.4	0.507877	0.52986

CWSI	Ig Observed from graph T3	Ig Predict from the Equation T3
0.4	0.534	0.518
0.5	0.476	0.471
0.6	0.395	0.424

CWSI	Ig Observed from graph T4	Ig Predict from the Equation T4
0.3	0.562	0.646
0.4	0.492	0.583
0.5	0.492	0.521

## Discussions

This research utilized a drone to capture thermal images of plant leaves and extract temperature data. The results demonstrated that thermal imaging effectively distinguished between different treatments. The drone facilitated smooth thermal image acquisition compared to sensors placed directly on the leaf. This finding aligns with Romero et al.^35^, who demonstrated that thermal imaging can differentiate between stressed and unstressed vines.

This study also showed that fluctuating water levels (both high and low) can negatively impact plant health. This was evident in T2 and T3, where high water content initially showed no visual effects, but over time, plants became stressed. Evaluating the correlations between canopy temperature and meteorological variables such as air temperature, relative humidity, VPD, and solar radiation revealed that increased water availability can lower canopy temperature and vice versa. The canopy temperatures (Tc) across the four treatments varied throughout the study period, with their range and standard deviation (SD) calculated and presented in Table 5.

**Table 5:** Daily range (R), standard deviation (SD), and air vapor pressure deficit (VPD) of canopy temperature (Tc) for each treatment.

Date	Treatment	TC range (°C)	VPD (Kpa)	STD
30-Jan	T1	11.41	0.12	3.57
	C	11.05		3.65
	T2	9.90		3.30
	T3	8.49		3.20
15-Feb	T1	8.75	0.11	2.65
	C	8.02		2.34
	T2	7.92		2.39
	T3	7.24		2.18
15-Mar	T1	11.74	0.13	3.60
	C	11.71		3.51
	T2	11.26		3.45
	T3	11.02		3.36
1-Apr	T1	14.31		4.15
	C	13.25		3.85
	T2	12.93	0.15	3.56
	T3	12.52		3.34

Greater variations result in more significant standard deviations. As soil moisture decreases, the canopy temperature’s daily range (*R*) and SD increase, with T1 exhibiting the highest variation. Water availability to plants also varied proportionally under the same conditions. This study indicated that a higher air VPD led to higher R and SD values, particularly in the hotter months (April and March) compared to the colder months (January and February). Lean et al.^36^ confirmed that VPD and soil moisture influence Tc variability.

The sensitivity of canopy temperature, CWSI, and relative stomatal conductance under various irrigation regimens and greenhouse weather conditions, as shown in Figs. 10 and 14, provides valuable insights into plant physiological responses to water stress. Canopy temperature and CWSI are especially sensitive indicators as they directly reflect variations in transpiration and water availability; higher values generally indicate greater stress. By measuring stomatal control of water loss, which fluctuates with irrigation and environmental conditions including temperature, humidity, and solar radiation, relative stomatal conductance complements these indicators. A highly effective tool for monitoring these indicators is drone-based thermal imaging technology, which enables rapid, extensive, and noninvasive assessment of plant water status. Its ability to integrate geographical variability with high-resolution thermal data for plant canopy temperature enhances precision irrigation.

CWSI values reliably predict plant-available water in peppers, making them useful for assessing plant status when CWSI is known. Researchers have found a strong correlation between canopy temperature and plant water stress^34^. García-Tejero et al.^37^ concluded that CWSI is best evaluated at midday, between 12:00 and 13:00, while Bellvert et al.^38^ established a reliable approximation for well-watered leaves’ CWSI between 10:00 and 16:00 h. This study analyzed pepper plant water stress levels using canopy temperatures extracted from thermal imagery across different treatments, aggregating data at midday. The CWSI results align with previous research^39,40^, indicating that under severe water deficiency, CWSI values approach 1.0, while in fully irrigated conditions, they remain below 0.5.

Based on these results, the calculated CWSI and Ig demonstrate their impact on plant physiological processes. Plants subjected to under-irrigation regimes exhibited high CWSI values, with stomata partially or fully closing to minimize water loss, sequentially reducing CO₂ uptake and photosynthesis rates. As transpiration decreases with higher CWSI, plants conserve water, altering the surrounding microclimate. This also limits water availability for light-dependent reactions, reducing photosynthetic efficiency. High CWSI correlates with increased leaf temperature due to reduced transpiration. Conversely, stomatal conductance plays a crucial role in processes such as photosynthesis and transpiration, as confirmed by Dong et al.^41^. Iqbal et al.^42^ and Ramos-Fernández et al.^43^ demonstrated that most plants reduce stomatal conductance in response to declining soil water availability. Grossiord et al.^44^ confirmed that plants close their stomata when soil moisture decreases, slowing water evaporation and increasing canopy temperature findings consistent with this study.

Moreover, the results indicate that in the context of “good watering” or proper irrigation, crops receive enough water to maintain relatively high soil moisture levels, reducing plant stress. Consequently, a lower CWSI often denotes healthier, less stressed crops. Studies by Xu et al.^45^ have shown an inverse relationship between soil water content and CWSI values. When the soil has sufficient water, the CWSI decreases, indicating minimal plant stress, and vice versa. As observed in our study, T1, which had lower water availability, exhibited a high CWSI, as shown in Fig. 10, making it a valuable indicator of crop water stress levels for irrigation management. In summary, CWSI influences soil characteristics and crop physiological, biochemical, and ecological responses, primarily reflected in reduced transpiration rates, which further impact crop canopy temperature, as noted by Dong et al.^41^.

In this study, temperature variations between canopies under different irrigation treatments were analyzed using drone-based thermal photography with attached thermal sensors to assess plant health. These heat indices (CWSI and Ig) are generally effective in detecting relative stress levels necessary for irrigation scheduling. This suggests that as water stress increases, stomatal conductance decreases. The study by Ramos-Fernández et al.^43^ confirms that, when combined with UAV-captured images, this technology serves as an effective tool for monitoring and managing water stress in field-grown rice crops, with potential benefits for greenhouse conditions, as tested and validated by our study.

Abscisic acid (ABA) is produced in roots under drought conditions and is transduced to the aerial parts of the plant through transpiration, where it regulates leaf physiological functions such as the leaf expansion rate and stomatal conductance^46^. However, severe water stress can damage leaf tissue and reduce photosynthesis^47^. According to Pérez-Pérez et al.^48^, drying specific root zone areas may trigger ABA production in roots and ethylene emission from leaves, reducing transpiration, stomatal opening, and leaf growth rate, as noted by Chai et al.^49^. Conversely, over-irrigation increases ethylene production, which promotes aerenchyma formation for oxygen transport but may also accelerate senescence and cause leaf wilting^50^. Genes related to anaerobic metabolism and ethylene signaling are highly expressed in over-irrigated plants, activating molecular pathways that induce responses similar to hypoxia stress. This behavior was observed in plants subjected to over-irrigation in our experimental study.

Our study highlights several benefits of using drones with cameras to monitor plant health within greenhouses, including enhanced flexibility, efficiency, high-resolution data collection, and comprehensive thermal imaging coverage based on canopy temperatures. Drones provide a more dynamic and thorough solution, making them particularly valuable for precise and timely plant health monitoring, especially in arid and challenging environments where manual monitoring is more labor-intensive. In contrast, traditional sensor-based systems provide continuous data from specific locations but may alter the canopy. Additionally, this study found a high correlation between thermocouple and thermal camera measurements, indicating their consistency. However, drones equipped with cameras offer greater efficiency due to their flexibility compared to sensor-based drones, which require frequent checks to ensure the thermocouple sensor is correctly attached to the canopy.

## Conclusion

This study used pepper plants in a CEA to detect canopy temperature (Tc) variation under different irrigation treatments. A drone-attached sensor was employed to detect Tc, potentially addressing limitations in plant health monitoring. Four irrigation regimes (T1, T2, T3) along with the control treatment (C) were used to evaluate the effectiveness of a thermal camera mounted on the drone. Tc values from thermal images taken at midday showed a lower R^2^ in the morning and evening due to illumination differences. The overall correlation between the sensor-based and drone-based thermal sensors used in this study to detect Tc was excellent, reaching an R^2^ value of 0.959. Plant water status was assessed by monitoring Tc data from thermal images through CWSI. CWSI was closely related to the relative stomatal index (Ig), which can guide crop health by distinguishing between stressed and unstressed plants. The C treatment showed a significant difference in Tc compared to all other treatments, and the calculated CWSI exhibited a high correlation.

This study established a relationship between the indicators Ig and CWSI, where a decrease in Ig led to an increase in CWSI. The thermal-based CWSI developed in this study requires no additional information and may be sufficient for detecting relative stress levels necessary for irrigation scheduling. The objectives of this work were to optimize the drone-attached thermal camera for detecting thermal imaging to determine plant responses to water deficits in CEA and to evaluate thermal imaging as a tool for distinguishing between stressed and unstressed plants. Experiments were conducted to test whether thermal imaging could differentiate irrigated and water-limited pepper plants under different irrigation treatments. The relationship between canopy and leaf temperatures, as well as the thermal indices derived from these temperatures, was investigated.

This technology would help growers with the early detection and management of plant health issues, representing a shift toward more sustainable, efficient, and resilient agricultural practices that do not rely on traditional arable lands. Finally, we believe this study will open new avenues for research to include other crops and irrigation regimes in the future. To broaden the scope of such studies, we recommend expanding research to incorporate more crops and irrigation treatments under different growth stages.

## Data Availability

The set of data generated and/or analyzed during the present study are available through the corresponding author upon reasonable request.
